# Complete Genome Sequences of Escherichia coli Phages vB_EcoM-EP75 and vB_EcoP-EP335

**DOI:** 10.1128/MRA.00078-19

**Published:** 2019-04-18

**Authors:** Joël van Mierlo, Steven Hagens, Sander Witte, Silvia Klamert, Leoni van de Straat, Lars Fieseler

**Affiliations:** a Micreos Food Safety, Wageningen, The Netherlands; b Food Microbiology Research Group, Institute of Food and Beverage Innovation, Zurich University of Applied Sciences (ZHAW), Wädenswil, Switzerland; Queens College

## Abstract

Phages vB_EcoM-EP75 (EP75) and vB_EcoP-EP335 (EP335) specifically infect Shiga toxin (Stx)-producing *Escherichia coli* (STEC) O157 strains. EP75 has a genome size of 158,143 bp and belongs to the genus Vi1virus. The genome size of EP335 is 76,622 bp, and it belongs to the genus Phieco32virus.

## Abstract (precis)

Phages vB_EcoM-EP75 (EP75) and vB_EcoP-EP335 (EP335) specifically infect Shiga toxin (Stx)-producing *Escherichia coli* (STEC) O157 strains. EP75 has a genome size of 158,143 bp and belongs to the genus Vi1virus.

## ANNOUNCEMENT

Shiga toxin (Stx)-producing Escherichia coli (STEC) strains are a tremendous threat to human health, as they cause severe infections and kidney failure (1). Some antibiotics cannot be applied to control the disease either because the bacteria are resistant to the antibiotic or because the expression of Shiga toxins is upregulated after antibiotic exposure (1). To control these pathogenic bacteria, bacteriophage treatment represents one of the alternatives to antibiotics (1). Importantly, treatment using bacteriophages does not seem to induce expression of Shiga toxins (2). Although more research is needed before bacteriophages can be applied in human therapy, the application of bacteriophages in food is already industry practice. This creates the opportunity to exploit bacteriophages as an intervention against STEC in food. To serve this purpose, we have set out to isolate and sequence STEC O157-specific bacteriophages.

STEC O157-specific phages vB_EcoM-EP75 (EP75) and vB_EcoP-EP335 (EP335) were isolated from sewage water from The Netherlands using the LB soft agar overlay method (3) and E. coli O157 NC13127 as an isolation strain. Plates were incubated overnight at 37°C (EP75) or 20°C (EP335). Single plaques were picked using a Pasteur pipette and transferred to 300 µl of sodium chloride-magnesium sulfate (SM) buffer (100 mM NaCl, 8 mM MgSO_4_, 50 mM Tris-HCl [pH 7.4]), followed by a 2-h incubation at 30°C. After centrifugation, the supernatant was filter sterilized (0.2-µm membrane filter) and used in two consecutive rounds of plaque isolation to obtain a pure phage solution. Filter-sterilized lysates (0.2-µm membrane filter) of EP75 and EP335 were precipitated using polyethylene glycol (10% [wt/vol] PEG 8000, 1 M NaCl; 4°C, overnight) and pelletized (10 min, 10,000 × *g*), after which the pellet was resuspended in 5 ml of SM buffer. Phages were purified using CsCl gradient centrifugation (4).

Phage DNA was extracted using the Quick-DNA viral kit (Zymo Research, Irvine, CA) according to the manufacturer’s protocol. Library preparation was performed using the Nextera XT library preparation kit (Illumina, San Diego, CA) according to the manufacturer’s instructions. The resulting paired-end sequence reads were generated using an Illumina HiSeq 2500 system. Sequencing of the EP75 genome revealed read lengths of >50 nucleotides and a total of 3,491,911 reads, with an average quality (Phred) score of 37.82. Sequencing of the EP335 genome revealed read lengths of >50 nucleotides, a total of 589,508 reads, and an average quality (Phred) score of 38.78. For read quality control, FASTQ sequence files were generated using bcl2fastq2 version 2.18. Initial quality assessment was based on data passing Illumina Chastity filtering. Subsequently, reads containing the PhiX control signal were removed using an in-house filtering protocol. In addition, reads containing adapters were clipped. The second quality assessment was based on the remaining reads, using the FASTQC version 0.11.5 quality control tool. Reads of the EP75 genome were assembled into 8 contigs using SPAdes version 3.10 (5). For the EP335 genome, reads were assembled into 15 contigs. BLASTN 2.8.1+ (default settings) was used to search for the closest matches for all contigs. Contigs were placed into scaffolds using the SSPACE Premium Scaffolder version 2.3 (6). Contigs with more than 50% coverage and 80% sequence identity to non-phage-related sequences were discarded for genome analysis. The average coverages of the assembled genomes were 5,129 (EP75) and 1,664 (EP335) reads per nucleotide.

Coding sequences (CDS) were annotated using Rapid Annotations using Subsystems Technology (RAST) 2.0 (7) and BLAST (8) comparisons with the nonredundant GenBank database. ARAGORN version 1.2.38 (9) and tRNAscan-SE version 2.0 (10) identified tRNA sequences. Overall nucleotide sequence identities were analyzed using EMBOSS Stretcher (11) and BLAST2seq.

The EP75 genome is 158,143 bp long and likely circularly permuted (12). The G+C content is 44.6%. Of the 209 CDS annotated, 57 were assigned a putative function. Five tRNA sequences were found. EP75 shares a nucleotide identity of 96.59% with *Escherichia* phage PhaxI (query coverage, 91%; GenBank accession number JN673056), 92.8% with *Salmonella* phage Marshall (query coverage, 86%; accession number KF669653), and 91.92% with *Salmonella* phage Maynard (query coverage, 85%; accession number KF669654), placing EP75 into the family Ackermannviridae, subfamily Cvivirinae, and genus Vi1virus. With nucleotide identities ranging from 91.92 to 95.69% and query coverages ranging from 85 to 90%, EP75 also exhibited homologies to Escherichia phage ECML-4 (GenBank accession number JX128257), Salmonella phage 38 (accession number KR296692), and *Escherichia* virus CBA120 (accession number JN593240).

The double-stranded linear DNA of EP335 is 76,622 bp long with a G+C content of 42.5% and contains 125 annotated CDS, 20 with assigned putative functions, and a tRNA^Arg^ sequence. Due to the nucleotide identity of 96.73% with *Escherichia* phage KBNP1711 (query coverage, 88%; GenBank accession number KF981730) and 85.47% with *Escherichia* phage NJ01 (query coverage, 69%; accession number JX867715), EP335 can be assigned to the family Podoviridae, genus Phieco32virus. Compared to other phages, EP335 exhibited homology to *Escherichia* phage 172-1 (accession number KP308307), Enterobacteria phage phiEco32 (accession number EU330206), and phage vB_EcoP_SU10 (accession number KM044272), with nucleotide identities of ca. 85% and query coverages ranging from 65 to 70%.

### Data availability.

The annotated sequences of the two E. coli phage genomes were deposited at GenBank under the accession numbers MG748547 (vB_EcoM-EP75) and MG748548 (vB_EcoP-EP335). The raw data are available under BioProject and SRA accession numbers PRJNA517780 and SRP182885, respectively.
